# Transforming growth factor beta (TGFβ) signaling is regulated at the pocket region of primary cilia

**DOI:** 10.1186/2046-2530-1-S1-O23

**Published:** 2012-11-16

**Authors:** S Christensen, CA Clement, SK Brorsen, KD Ajbro, M de Jesus, LB Pedersen, LA Larsen

**Affiliations:** 1 Department of Biology, University of Copenhagen, Denmark; 2 Wilhelm Johannsen Centre for Functional Genome Research, University of Copenhagen, Denmark

## 

TGFβ signaling extensively cross-talks with Hh, Wnt and RTK signaling to control cell proliferation, migration and differentiation, and when aberrantly regulated leads to developmental defects and cancer. TGFβ signaling is activated through the internalization of TGFβ receptors via clathrin-dependent endocytosis (CDE), at which the receptor activates Smad transcription factors. Here we investigated the relationship between TGFβ signaling and primary cilia in fibroblasts and in EC and human embryonic stem cells during their differentiation into cardiomyocytes and neurons using transcriptomics, imaging and molecular biology tools. During cardiomyocyte differentiation, expression of TGFβ receptors and Smad proteins were up-regulated and targeted to the pocket region of primary cilia, at which the receptor colocalized with clathrin-coated pits and vesicles to activate Smad2/3. This activation was blocked by receptor antagonists or by *Ift20* knockdown. In contrast, neuronal differentiation was associated with a loss of ciliary TGFβ signaling. In mouse embryonic fibroblasts (MEFs) and human foreskin fibroblasts (hFFs), TGFβ stimulation increased the targeting of TGFβ receptors to the ciliary pocket region followed by activation of Smad signaling to promote cell cycle entry. In *Tg737orpk* MEFs there was a major reduction in TGFβ-induced Smad2/3 phosphorylation, and this was associated with reduced activity of clathrin-dependent endocytosis at stumpy primary cilia. Similarly, inhibition of CDE blocked activation of Smad2/3 at the ciliary pocket region in hFFs. Our results suggest that the ciliary pocket region functions as a unique site for regulation of TGFβ signaling and potentially in cross-talking with other signaling pathways during development and in tissue homeostasis.

